# Ambient air pollution and prostate cancer risk in a population-based Canadian case-control study

**DOI:** 10.1097/EE9.0000000000000219

**Published:** 2022-07-19

**Authors:** Leslie Michele-Ange Kouam Youogo, Marie-Elise Parent, Perry Hystad, Paul J. Villeneuve

**Affiliations:** a Department of Neuroscience, Carleton University, Ottawa, Ontario, Canada; b University of Bordeaux, ISPED, Bordeaux, France; c Epidemiology and Biostatistics Unit, Centre Armand-Frappier Santé Biotechnologie, Institut national de la recherche scientifique, Université du Québec, Laval, Quebec, Canada; d Department of Social and Preventive Medicine, School of Public Health, Université de Montréal, Montreal, Quebec, Canada; e Centre de recherche du CHUM, Montreal, Quebec, Canada; f College of Public Health and Human Sciences, Oregon State University, Corvallis, Oregon

**Keywords:** Air pollution, Case-control study, Fine particulate matter, Nitrogen dioxide, Prostate cancer

## Abstract

Ambient air pollution is a human carcinogen and a possible risk factor for prostate cancer.

**Methods:**

We investigated associations between ambient concentrations particulate matter 2.5 (PM_2.5_) and nitrogen dioxide (NO_2_) and incident prostate cancer in a Canadian case-control study. Between 1994 and 1997, cases were identified from provincial cancer registries, and a population-based series of controls was recruited. Among men 50 years of age or older, risk factor and residential history data (1975 to 1994) were collected from 1,420 prostate cancer cases and 1,424 controls. Three methods were used to estimate the residential mean exposure to PM_2.5_ and NO_2_ during this period: (1) satellite-derived observations; (2) satellite-derived observations scaled with historical fixed-site measurements; and (3) a national land-use regression (LUR) model. Odds ratios (ORs) and their 95% confidence intervals (CIs) in relation to interquartile range (IQR) increases in PM_2.5_ and NO_2_ were estimated using logistic regression, adjusting for personal and contextual factors.

**Results:**

We found positive associations between exposure to PM_2.5_ and NO_2_ over the previous 20 years and prostate cancer. An IQR increase in PM_2.5_ (3.56 µg/m^3^ for satellite and 4.48 µg/m^3^ for scaled satellite observations) yielded ORs of 1.28 (95% CI = 1.07, 1.52) and 1.20 (95% CI = 1.03, 1.40), respectively. For NO_2_, IQR increases (1.45 ppb for satellite, 15.18 ppb for scaled satellite-derived information, and 15.39 ppb for the national LUR) were associated with ORs of 1.09 (95% CI = 0.95, 1.24), 1.21 (95% CI = 1.02, 1.43), and 1.19 (95% CI = 1.03, 1.38), respectively.

**Conclusions:**

Our findings support the hypothesis that ambient air pollution increases the risk of prostate cancer.

**What this study adds**

Prostate cancer remains an important public health problem because it is one of the most commonly diagnosed cancer in men. In this study, we examined the potential role of outdoor air pollution in its etiology. This study found that exposure to NO_2_ and PM_2.5_ was associated with an increased risk of prostate cancer. The findings presented in this article fill an important research gap as few studies have previously evaluated associations between air pollution and prostate cancer. This study adds to the growing epidemiological literature that exposure to ambient air pollution may increase the risk of nonrespiratory cancers.

## Introduction

Prostate cancer is the second most commonly diagnosed cancer and the fifth leading cause of cancer death in men worldwide.^1^ In 2021, there were an estimated 24,000 new cases of prostate cancer and 4,500 deaths in Canada.^2^

Currently, age, ethnicity, and family history of prostate cancer are the only established risk factors for prostate cancer, but these are not modifiable.^3^ The role of several modifiable risk factors has been evaluated but the findings have been inconsistent. These factors include lifestyle (diet, physical activity, anthropometry) and occupational exposures (e.g., cadmium, pesticides).^4,5^ Studies of prostate cancer in migrants and as well as observed geographical variations in prostate cancer incidence rates suggest that environmental exposures are etiologically relevant.^6^

Ambient air pollution is characterized by the contamination of the environment by chemical, physical, or biological agents. The most common sources are emissions from motor vehicles, combustion processes of solid fuels, and industry.^7^ Fine particles with an aerodynamic diameter of less than 2.5 µm (PM_2.5_) and nitrogen dioxide (NO_2_) are among the most commonly studied air pollutants. NO_2_ is a marker for vehicle emissions and contain carcinogens such as polycyclic aromatic hydrocarbons (PAHs) and benzo(*a*)pyrene.^8^ Air pollution is a recognized risk factor for cardiovascular and respiratory diseases, and in 2013, the International Agency for Research on Cancer (IARC) classified particulate matter air pollution as a human carcinogen.^9–11^ While there is sufficient evidence of an association between air pollution and lung cancer, the evidence for other cancer sites is limited. The possibility that air pollution increases the risk of developing nonrespiratory cancers is strengthened with findings of positive associations for brain, colorectal, and breast cancer.^12–14^ For prostate cancer, few studies have assessed the impact of air pollution on incidence and the findings have been mixed.^15–19^ There are several plausible mechanisms whereby exposure to air pollution increases the risk of prostate cancer. Air pollution-related chemicals can potentially act directly on the prostate through cell membrane disruption, induction of pro-inflammatory cytokines and tumor necrosis factor α, and apoptosis by the mitochondria; in addition, PAHs, benzene, and cadmium can act indirectly through endocrine-disrupting properties.^20,21^

Given the relatively few studies on air pollution and prostate cancer and the inconsistency in their findings, we sought to evaluate associations between past long-term exposure to ambient NO_2_ and PM_2.5_ using a previously conducted Canadian population-based case-control study.

## Methods

### Study design

Our study population was drawn from the Canadian National Enhanced Cancer Surveillance System (NECSS). The NECSS is a large multisite cancer population-based case-control study conducted between 1994 and 1997. Data were collected from participants who resided in eight of the 10 Canadian provinces (all provinces but Quebec and New Brunswick). The overall aim of the NECSS was to identify environmental causes of cancer. Detailed methods for the NECSS are published elsewhere.^22^ Briefly, in this population-based case-control study, incident cancer cases were identified by provincial cancer registries. The NECSS included cancer cases for 19 different types of cancer sites. All cancers were histologically confirmed diagnoses of cancer and classified using the International Classification of Diseases, 9th revision (ICD-9) rubrics.^23^ Controls were selected during the same time period as the cases from a random sample of individuals from each of the participating provinces, the age and sex distribution of controls in the NECSS was frequency-matched to the age-sex distribution across all 19 types of cancers. For age, frequency matching was done by 5-year age groupings. The control selection strategy varied by province depending on data availability and accessibility: Provincial health insurance plans (95% of individuals are covered by this type of service; however, military personnel and their families and Aboriginals were excluded because they are covered by other plans) were used in British Columbia, Saskatchewan, Manitoba, Prince Edward Island, and Nova Scotia; In Ontario, controls were selected using data from the Ministry of Finance Property Assessment, whereas in Alberta and Newfoundland, random digit dialing was used.

The NECSS sought the participation of 2,781 histologically confirmed incident prostate cancer cases (ICD-9: 185) that were initially identified. Of these, 414 men were excluded from the study for any of the following reasons: patient had died, incorrect mailing address, or physician consent was not granted. Self-administered surveys were mailed to the remaining 2,367 men, and of these, 1,799 were completed and returned, representing a 76% participation rate. Questionnaires were mailed to 4,235 men identified as potential controls. For 287 of these men (6.8%), questionnaires were returned indicating an incorrect address. Among controls, 2,547 men completed and returned the questionnaire representing a 64.5% participation rate.

We restricted the analysis to cases and controls 50 years of age and older as diagnoses of prostate cancer at younger ages are rare and tend to reflect genetic susceptibility rather than an environmental cause.^24^ This was a relatively small number of cases (n = 12).

### Data collection

Participants were asked to provide demographic information including age, ethnicity, socioeconomic status (SES), education, and residential history. The questionnaire also asked participants to provide anthropometric measures (height and weight), as well as information on their smoking history, exposure to environmental tobacco smoke, alcohol consumption, physical activity, and dietary patterns. Self-reported measures of exposure to pesticides and cadmium were also collected. A complete residential history (since the age of 18) was sought, and participants were asked to provide their address, including their six-character postal code.

### Exposure assessment to air pollution

Based on residential histories, an average air pollution exposure concentration was assigned to each participant for each year between 1975 and 1994. We restricted analyses to subjects with 20 years of exposure in the main analyses. Exposures were derived by a member of the team (P.H.) and details about the methodology that was used have previously been published.^25^ In brief, this exposure characterization involved first geocoding the six-character postal codes from the residential histories using the 1996 DMTI Spatial, Inc (Markham, Ontario, Canada). postal code database. In Canadian urban areas, a six-character postal code represents a specific block (one side of a street between two intersecting streets), a single building or sometimes a large volume mail receiver, while in rural areas, a 6-character postal code represents larger areas, sometimes an entire town or county.^26,27^ After geocoding the postal address’s location to assign the NO_2_ and PM_2.5_ concentrations of the subjects for all addresses they lived at during the study period, we assigned pollution concentrations using three methods. We opted to model three different exposure metrics as these measures have distinct strengths and limitations. For example, our satellite measurements were able to cover large geographical areas including those not covered by ground-based fixed-site monitors. The satellite measures could also be back-extrapolate to provide historical measures of air pollution concentrations. However, the satellite-derived exposure metric was limited in that the spatial resolution was modest (10 × 10 km). the land-use regression (LUR) model, on the other hand, provided a highly spatially resolved surface that could better capture smaller within-city variations in exposure but lacked the temporal component. Our decision to model these three metrics allowed us to better understand how the temporal and spatial components of these exposures impacted disease risks. The metrics are described in more detail below.

#### Satellite measures of PM_2.5_ and NO_2_

NO_2_ and PM_2.5_ concentrations were estimated from satellite measurements (using 2005–2007 retrievals for NO_2_ and 2001–2006 retrievals for PM_2.5_) at 10 × 10 km resolution, scaled with a chemical transport model to estimate ground-level concentrations.^28,29^

#### Scaled measures of PM_2.5_ and NO_2_

To account for long-term spatiotemporal trends in air pollution between 1975 and 1994, we estimated the annual air pollutant concentrations during this period using satellite data that were calibrated using information from the National Air Pollution Surveillance (NAPS) network.^30^ The calibration was carried out as follows:

NAPS monitoring data were first organized into monthly averages for both NO_2_ and PM_2.5_. Continuous monitoring data were included if at least 50% of the daily hourly observations were available and at least 50% of days were available in a month. Annual averages were calculated when complete data were available for at least 6 months with at least one month per season. Given the lack of spatial and temporal measurements of PM_2.5_ before 1984, collocated measurements of PM_2.5_ and total suspended particles (TSPs) between 1984 and 2000 were used to create predictive models of historical PM_2.5_ concentrations: random effects linear regression models (GLIMMIX procedure in SAS 9.3, Cary, North Carolina) were developed to account for the clustering of annual measurements over time at each NAPS monitoring station. The *R*^2^ and root mean square error (RMSE) were 0.67 and 2.31, respectively; the resulting model was applied to all valid TSP monitoring stations. Next, at each ground-based fixed monitoring site, a ratio of annual measured concentration of NO_2_ and PM_2.5_ to the satellite estimated concentration was computed, and then we conducted smoothed inverse distance weighting interpolation of the ratios. The resulting interpolated areas were multiplied by the original satellite area, generating histories of the adjusted annual NO_2_ and PM_2.5_ estimates for each year from 1975 to 1994.

#### Land-use regression surface for NO_2_

For the third method (LUR surface for NO_2_), data were provided by the Canadian Consortium on Urban Environmental Health (CANUE: www.canue.ca). Details of the method used to generate these data are provided elsewhere.^31^ Briefly, the national NO_2_ LUR model was developed using 2006 NAPS fixed-site monitoring data. To capture background and regional NO_2_ variation, NO_2_ satellite estimates from 2005 to 2011, area of industrial land use within 2 km, road length within 10 km and summer rainfall were used as predictors. To capture local-scale variations, distance-decay gradients were developed from a literature review and applied to the regression model estimates for postal codes near highways (NO_2_ increases around 65% near highways and decreases to background levels at 300 m) and major roads (NO_2_ increases around 20% near major roads and decreases to background level at 100 m). The final LUR model explained 73% of the variation in NAPS measurements with a RMSE of 2.9 ppb and matches well with city-specific NO_2_ land-use regression models, which typically have *R*^2^ values between 0.54 and 0.81.^32^ The model was developed for all study postal codes to estimate concentrations of NO_2_, and we then calculated an average concentration for the 1975–1994 exposure period; NO_2_ concentrations for year 1984 were assigned to years prior to 1984.

#### Roadways measures

We assessed proximity to roadways as an alternative surrogate measure of vehicle emissions. Due to the lack of historical data on the national road network, we used the 1996 road network data (DMTI Spatial, Inc.); from this, we estimated estimate the number of years lived within 50,100 and 300 m of roads (highway and/or major road). This variable was transformed into a two-category variable: subjects who never lived near these roads and the others who lived near either or both.

### Covariates

Individual characteristics collected from the survey were used to derive a series of adjustment factors for inclusion in our models, including: ethnicity (Caucasian, Black, Asian, and other), alcohol consumption (servings/week), smoking (pack-years), physical activity (number of hours per month of moderate and strenuous activity), total caloric intake (derived from a food-frequency questionnaire about recent intakes), body mass index (BMI) (<20, 20 to <25, 25 to <30, and ≥30 kg/m^2^), self-reported exposure to pesticides and cadmium at home or at work (never/ever), and level of education and residential surrounding greenness.^33–35^ The latter was based on the Normalized Difference Vegetation Index (NDVI), which is a commonly used measure of vegetation. Our measure of NDVI covered a 500 m buffer around the residence for 20 years (between 1975 and 1994). The neighborhood socioeconomic status index (in quintiles) was also considered as a potential confounder as it has been shown that less affluent neighborhoods have, on average, higher concentrations of air pollution^36,37^; moreover, men who live in a socially advantaged neighborhoods have been found to be greater risk of being diagnosed with prostate cancer.^38^

### Statistical analysis

We used (unconditional) logistic regression to estimate odds ratios (ORs) and their 95% confidence intervals (CIs) of associations between exposure to air pollution and prostate cancer. We modeled these association in relation to an interquartile range (IQR) increase in NO_2_ (1.45 ppb, 15.18 ppb, and 15.39 ppb for satellite, scaled, and LUR, respectively) and PM_2.5_ (3.56 µg/m^3^ and 4.48 µg/m^3^ for satellite and scaled satellite, respectively). This allowed us to compare the strength of the association across the different pollutants and metrics. For all analyses, three different models were fit:

- Model 1: included only age and province of residence at the time of completing the survey.
- Model 2: same variables as model 1 and additional personal potential confounders: ethnicity, BMI, alcohol consumption, total caloric intake, education, moderate and strenuous physical activity, smoking, exposures to pesticide and cadmium.
- Model 3: same variables as model 2 with the addition of two contextual variables: a long-term neighborhood socioeconomic status index and residential surrounding greenness.

Age, smoking, education, total calorie intake, alcohol consumption, moderate and strenuous physical activity, and greenness were modeled as continuous variables after confirming linearity of the logits. We assessed the shape of the exposure-response function between exposure to pollutants and prostate cancer by using a cubic smoothing splines function with 4 degrees of freedom (*df*).^39^

Due to the presence of missing data for some covariates (ranging from 0.13% to 21.1%) and assuming that the mechanism behind them was either Missing Completely at Random (MCAR) or Missing at Random (MAR), we conducted multiple imputations using chained equations (MICE).^40^ The main results presented herein are based on analyses that used imputation.

### Sensitivity analyses

In addition to the main analyses, we performed sensitivity analyses by restricting to participants with complete data to evaluate the robustness of the results when compared to findings based on imputed data. Because of postal codes in rural areas are not highly spatially resolved, we also performed separate analyses restricted to subjects living in urban areas (which corresponds to an area with a population of at least 1,000).^41^

## Results

The original NECSS data set included 4,346 cases and controls; 1,502 subjects were excluded for one of the following reasons: missing address, age <50 years, and < 20 years of residential history between 1975 and 1994. As a result, a total of 2,844 subjects (1,420 cases and 1,424 controls) were included in our study (flow chart presented in Supplementary Material: Figure S1; http://links.lww.com/EE/A194).

**Figure 1.**
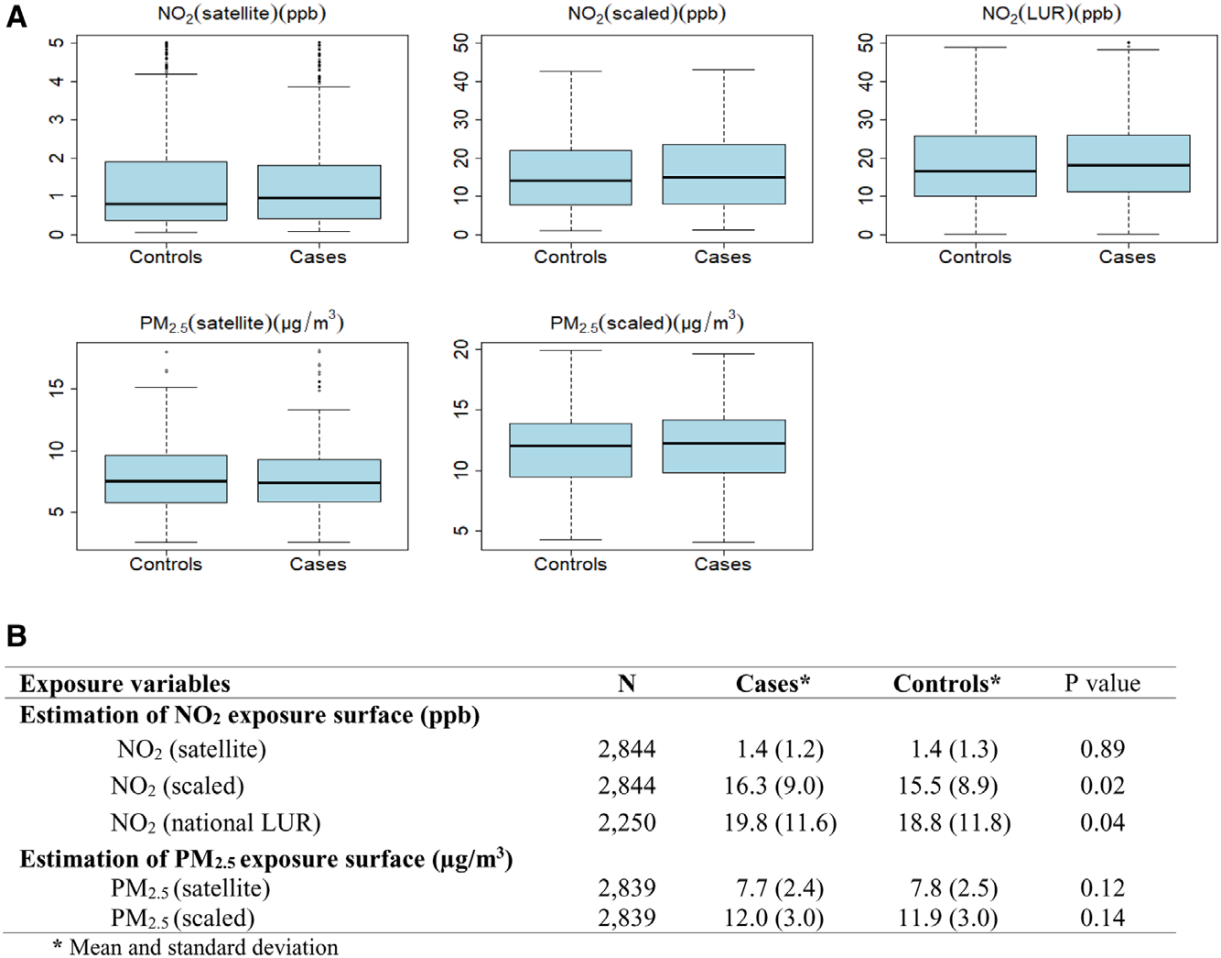
Comparison of air pollution concentrations between case and control series. Box plots comparing cases and controls data of air pollutant concentrations (A). Values shown are median (line within box), 25th and 75th percentiles (bottom and top of box, respectively). B, Air pollutant concentrations comparison data between cases and controls. NECSS, 1975–1994.

Table 1 shows the risk factor distributions for several characteristics among cases and controls. The average age at diagnosis in our case series was 66.7 years (±5.6 years), while the mean age in the control series was 65.5 years (±6.4 years). Study subjects were largely Caucasian, there was a slight difference in BMI distributions between cases and controls, pack-years were higher among controls than cases, and most cases were in the 4th and 5th (most favorable) quintile of the neighborhood SES index, while most controls were in the 1st and 3rd quintile. The distribution of pollutants concentrations varied by province with Ontario having the highest concentrations and Newfoundland the lowest. Figure 1 shows the distributions of NO_2_ and PM_2.5_ concentrations for cases and controls (The differences in the means were tested using the Student *t* test). The average 20-year concentration of pollutants was slightly higher for cases compared to controls: 1.4 ppb (±1.2) versus 1.4 ppb (±1.3); 16.3 ppb (±9.0) versus 15.5 ppb (±8.9); and 19.8 ppb (±11.6) versus 18.8 ppb (±11.8) for NO_2_ from satellite, scaled NO_2_, and NO_2_ from national LUR, respectively. For PM_2.5_ from satellite and scaled PM_2.5_, it was 7.7 µg/m^3^ (±2.4) versus 7.8 µg/m^3^ (±2.5) and 12.0 µg/m^3^ (±3.0) versus 11.9 µg/m^3^ (±3.0) in cases and controls, respectively.

**Table 1.** Individual characteristics of prostate cancer cases and population controls with 20 years of complete residential history from 1975 to 1994 (N = 2,844), NECSS, Canada

Variables	Cases (N = 1,420)	Controls (N = 1,424)
Province		
Alberta	205 (14.4)	176 (12.4)
British Columbia	436 (30.7)	188 (13.2)
Manitoba	88 (6.2)	96 (6.7)
Newfoundland	61 (4.3)	80 (5.6)
Nova Scotia	98 (6.9)	235 (16.5)
Ontario	401 (28.2)	514 (36.1)
PEI	63 (4.4)	45 (3.2)
Saskatchewan	68 (4.8)	90 (6.3)
Ethnicity/race		
Caucasian	1,372 (96.6)	1,330 (93.4)
Black	6 (0.4)	5 (0.4)
Asian	14 (1.0)	67 (4.7)
Other	21 (1.5)	14 (1.0)
Missing	7 (0.5)	8 (0.6)
Marital status		
Married, common law	1,243 (87.5)	1,235 (86.7)
Divorced/separated/single	126 (8.9)	121 (8.5)
Single	48 (3.4)	65 (4.6)
Other	0 (0.0)	1 (0.1)
Missing	3 (0.2)	2 (0.1)
Total household income		
< 10,000	32 (2.3)	38 (2.7)
10,000–19,999	147 (10.4)	149 (10.5)
20,000–29,999	252 (17.7)	235 (16.5)
30,000–49,999	373 (26.3)	363 (25.5)
50,000–99,999	253 (17.8)	250 (17.6)
> 100,000	47 (3.3)	51 (3.6)
Prefer not to respond	273 (19.2)	291 (20.4)
Missing	43 (3.0)	47 (3.3)
Income adequacy^a^		
Low income	190 (13.4)	200 (14.0)
Low middle income	258 (18.2)	251 (17.6)
Upper middle income	358 (25.2)	366 (25.7)
High income	280 (19.7)	252 (17.7)
Missing	334 (23.5)	355 (24.9)
Years of education, mean (SD)	11.4 (3.8)	11.4 (4.0)
Missing	18 (13)	16 (1.1)
Age, mean (SD)	66.7 (5.6)	65.5 (6.4)
Missing	2 (0.1)	4 (0.3)
Height (meter), mean (SD)	1.8 (0.1)	1.8 (0.1)
Missing	3 (0.2)	2 (0.1)
Body mass index (kg/m^2^)		
< 20	15 (1.1)	27 (1.9)
20–25	469 (33.0)	462 (32.4)
25–30	722 (50.8)	694 (48.7)
≥ 30	209 (14.7)	234 (16.4)
Missing	5 (0.4)	7 (0.5)
Fat consumption (g/week), mean (SD)	406.2 (240.3)	397.5 (198.3)
Missing	2 (0.1)	2 (0.1)
Total caloric intake (KJ/week), mean (SD)	62,483 (26,709)	60,256 (22,196)
Missing	2 (0.1)	2 (0.1)
Physical activity (hour/month)^b^, mean (SD)		
Moderate	19.4 (16.5)	17.7 (14.9)
Intense	8.5 (11.8)	7.3 (10.4)
Smoking pack-years, mean (SD)	23.9 (18.5)	26.1 (20.2)
Missing	19 (1.3)	35 (2.5)
Smoking status^c^		
Never	324 (22.8)	297 (20.9)
Former	865 (60.9)	840 (59.0)
Current	160 (11.3)	243 (17.1)
Missing	71 (5.0)	44 (3.1)
Alcohol servings/week, mean (SD)^d^	10.2 (12.3)	10.2 (12.5)
Self-reported exposure to pesticides		
Never	1,153 (81.2)	1,190 (83.6)
Ever	267 (18.8)	233 (16.4)
Missing	0 (0.0)	1 (0.0)
Self-reported exposure to cadmium		
Never	1,405 (98.8)	1,406 (98.9)
Ever	15 (1.1)	17 (1.2)
Missing	0 (0.0)	1 (0.0)
Surrounding residential greenness^e^	0.4 (0.1)	0.4 (0.1)
Missing	302 (21.3)	298 (20.9)
Residential area		
Urban	1,085 (76.4)	1,074 (75.4)
Rural	335 (23.6)	350 (24.6)
Neighborhood SES index^f^ (quintiles)		
≤ –0.341 (least favorable)	232 (16.3)	291 (20.4)
–0.341 to 0.119	255 (18.0)	268 (18.8)
–0.119 to 0.0793	248 (17.5)	273 (19.2)
0.0793 to 0.327	271 (19.1)	251 (17.6)
> 0.327 (most favorable)	277 (19.5)	245 (17.2)
Missing	137 (9.6)	96 (6.7)

^a^Calculated from household income and adjusted for the number of people in the household.

^b^Mean and SD were calculated only for physically active subjects.

^c^Mean and SD were calculated only for smokers (former and current smokers).

^d^Mean and SD were calculated only for drinkers.

^e^Mean and SD (see https://www.canuedata.ca/metadata.php).

^f^Long-term neighborhood socioeconomic status index (see Hystad et al., 2012).

PEI indicates Prince Edward Island.

### Association between prostate cancer and air pollution

The results from the three regression models for each exposure are shown in Table 2. For NO_2_, the model adjusted only for age and province (model 1) showed a modest association with prostate cancer risk regardless of the measure considered. In contrast, after adjustment for several personal factors (model 2), the scaled estimates and the national LUR estimates were indicative of elevated risks, with ORs of 1.20 (95% CI = 1.03, 1.41) for a 15.18 ppb increase in NO_2_ and 1.16 (95% CI = 1.01, 1.33) for a 15.39 ppb, respectively. Satellite surface-based risk estimates were weaker. Finally, for the fully adjusted model (model 3), there was a positive association for scaled estimates (OR = 1.21; 95% CI = 1.02, 1.43) and for national LUR estimates (OR = 1.19; 95% CI = 1.03, 1.38). Again, the strength of association with satellite surface estimates was weaker.

**Table 2.** Odds ratios in relation to an interquartile range increase in residential ambient concentrations of NO_2_ and PM_2.5_ over the period 1975 and 1994, and prostate cancer

Variables	N	Model 1^a^OR (95% CI)	Model 2^b^ OR (95% CI)	Model 3^c^ OR (95% CI)
NO_2_ exposure estimates (ppb) for an increase equal to the IQR^d^				
NO_2_ (satellite)	2,844	1.03 (0.92, 1.15)	1.10 (0.97, 1.23)	1.09 (0.95, 1.24)
NO_2_ (scaled)	2,844	1.07 (0.92, 1.25)	1.20 (1.03, 1.41)	1.21 (1.02, 1.43)
NO_2_ (national LUR)	2,250	1.08 (0.95, 1.23)	1.16 (1.01, 1.33)	1.19 (1.03, 1.38)
PM_2.5_ exposure estimates (µg/m^3^) for an increase equal to the IQR^e^				
PM_2.5_ (satellite)	2,839	1.23 (1.05, 1.45)	1.30 (1.10, 1.54)	1.28 (1.07, 1.53)
PM_2.5_ (scaled)	2,839	1.12 (0.98, 1.29)	1.22 (1.06, 1.40)	1.20 (1.03, 1.40)

^a^Model 1 adjusted for age and province.

^b^Model 2 adjusted for: age, province, ethnicity, pack-years, alcohol, BMI, years of education, moderate physical activity, strenuous physical activity, total caloric intake, exposure to pesticides, and exposure to cadmium.

^c^Model 3 adjusted for: model 2 variables, neighborhood SES index, and neighborhood greenness.

^d^IQR 1.45 ppb for satellite NO_2_, 15.18 for fused NO_2_, and 15.39 for national LUR NO_2_.

^e^IQR 3.56 ppb for satellite PM_2.5_ and 4.48 for fused PM_2.5_.

We observed positive associations between long-term PM_2.5_ exposure and prostate cancer incidence in all analyses except for scaled estimates in model 1. Using estimates of exposure from satellite, we found increased odds ratios of prostate cancer per 3.56 μg/m^3^, i.e., 1.23 (95% CI = 1.05, 1.45), 1.30 (95% CI = 1.10, 1.54), and 1.28 (95% CI = 1.07, 1.53) for models 1, 2, and 3, respectively. Using scaled PM_2.5_, for every 4.48 μg/m^3^ increase of PM_2.5_ concentration, the ORs were 1.12 (95% CI = 0.98, 1.29), 1.22 (95% CI = 1.06, 1.40), and 1.20 (95% CI = 1.03, 1.40) for models 1, 2, and 3, respectively.

The final model for NO_2_ and PM_2.5_ led to changes in the ORs, mainly related to the influence from the contextual variables of neighborhood socioeconomic status index and residential surrounding greenness.

**Figure 2.**
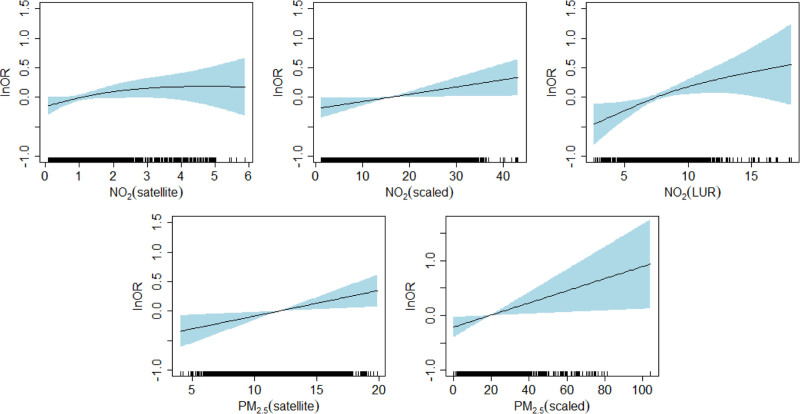
Exposure-response curves for the relationship between air pollutant concentrations (solid line) and 95% CI (blue shade) over the period from 1975 to 1994 and prostate cancer generated obtained using cubic smoothing splines at 4 df; fully adjusted model.

Figure 2 shows the fully adjusted (model 3) exposure-response function for each exposure evaluated for all historical residences from 1975 to 1994 (representations for models 1 and 2 are presented in a Supplementary Data: Figure S2a; http://links.lww.com/EE/A194 and Figure S2b; http://links.lww.com/EE/A194). Except for the satellite NO_2_ exposure-response for which the ORs increase up to a concentration of about 2 ppb, beyond which they remain stable, the exposure-response functions for other exposures were consistent with linearity and ORs increased proportionally to the concentration of the pollutant, with wider confidence intervals at the extremes due to the small number of subjects.

Regarding the proximity to roads, while no clear increase in odds was apparent with prostate cancer risk, the odds of prostate cancer tended to be somewhat higher among those who had ever lived near roads relative to those who had not (results for the analyses on imputed and complete data are presented in Supplementary Material: Table S1a; http://links.lww.com/EE/A194 and Table S1b; http://links.lww.com/EE/A194).

### Sensitivity analyses

Compared to the results on imputed data, after analysis on complete data, there were no material differences in the ORs between models 1 and 2. The adjusted odds ratios were, however, slightly lower for model 3 (see results in Supplementary Material: Table S2; http://links.lww.com/EE/A194). For an increase of an IQR, a positive association was observed for satellite observations of PM_2.5_ in all three models as well as for scaled PM_2.5_ estimates in model 2 (weaker in model 3). For NO_2_, a positive association was also observed for scaled estimates in model 2 and national LUR estimates in model 3 (weaker in model 2). For the analysis restricted to those who lived in urban area, the results on the imputed data showed that for an IQR increase, ORs tended to be higher than when rural subjects were included (results in Supplementary Material: Table S3; http://links.lww.com/EE/A194). For example, the fully adjusted ORs for NO_2_ were 1.10 (95% CI = 0.96, 1.26), 1.27 (95% CI = 1.05, 1.54), and 1.24 (95% CI = 1.07, 1.45) for satellite surface-based estimates, scaled estimates, and national LUR estimates, respectively. For PM_2.5_, the fully adjusted ORs were 1.31 (CI = 1.07, 1.60) and 1.21 (CI = 1.02, 1.44) for satellite surface-based estimates and scaled estimates, respectively.

## Discussion

Overall, we found that ambient NO_2_ and PM _2.5_ over a 20-year period were associated with an increased risk of prostate cancer. There have been few previous studies of this topic, and for two of these studies, prostate cancer was not the cancer of a priori interest.^18,19^ Previous studies typically considered only one pollutant and used different approaches to estimate long-term exposures. In our study, three approaches were used: satellite observations, which have the advantage of covering large geographical areas but whose nonoptimal spatial resolution limits the use, scaled satellite measurements (corresponding to the calibration of satellite observations with fixed sites of monitoring stations spread over the Canadian territory), which allowed for the temporal adjustment of satellite observations, and finally the national LUR model, which has a better spatial resolution and considers the local-scale variation of pollution, representing here the best approach to the estimation of the spatial variation of NO_2_.

Our positive findings are consistent with two other case-control studies of incident prostate cancer conducted in Montreal. The study by Parent et al^16^ focused on exposure to NO_2_ (in 2006 and 1996, i.e., 10 years before) measured using the LUR model. The other study by Weichenthal et al^15^ studied concentrations of ambient ultrafine particles (at the time of recruitment and 10 years before recruitment) measured using a LUR model. While we also found positive associations, the comparison of our findings to their is not straightforward due to differences in exposure periods, and methods to characterize exposure. Namely, we examined exposure over a period extending from 1975 to 1994, whereas these two studies examined exposure at recruitment and 10 years earlier. An ecological study carried out in the United States in Erie County (Pennsylvania) found an association between exposure to airborne particles (measured from monitors at different monitoring stations over a period of 2 years) and prostate cancer.^17^ Nevertheless, not all previous studies have reported positive associations. For example, a Danish cohort study of 57,053 subjects (men and women) recruited between 1993 and 1997 and followed up until 2006 that investigated the relationship between NO_2_ and 20 different types of cancers found an association between NO_2_ exposure and cervical and brain cancer but not with prostate cancer.^18^ To our knowledge, our study is the only one to report associations between proximity to roads and prostate cancer incidence. The lack of evidence for an association using this metric, contrasting with the others, may reflect the fact that distances to road metrics are less capable of capturing variations in exposure, such as due to traffic volume, than the other methods we used. Some studies have shown that agreement is not always good between air pollution measures from land-use regression surfaces and distance to roadway measures,^41^ and that LUR models are better predictors of traffic-related pollution.^42^

Our study has some limitations. Selection bias cannot be ruled out due to modest participation rates (76% for cases and 64.5% for controls) and exclusion of some subjects because they did not meet the inclusion criteria. However, it was observed that after adjusting for factors typically related to nonresponse (e.g., income, education), there were no appreciable changes in the risk estimates. This suggests the potential for participation bias is small.^43^ In addition, in our view, participation bias is likely minimal given that the risk estimates obtained from the NECSS data across a series of etiological risk factors for different cancer sites are similar to other published risk estimates. These risk factors have included diesel, obesity, and smoking, radon, and numerous others.^44–46^ Second, our analysis was limited due to missing covariates data. To mitigate this potential bias, we used multiple imputation, which represents a robust and valid approach for managing missing data and for obtaining unbiased estimators.^40^ Our sensitivity analyses suggest that there were no major differences in our measures of association between the imputed and nonimputed approaches. Third, the participants were not uniformly distributed geographically. However, the design of the NECSS study was such that each provincial cancer registry was responsible for sampling and recruiting study participants and a province variable was included in the models to capture any differences in sampling strategies. Fourth, residual confounding is possible in light of imperfectly measured or unknown confounders. Fifth, autopsy studies have shown that in men over 50 years of age, the prevalence of latent histological prostate cancer can be as high as 30%.^47,48^ Consequently, our controls likely included small proportion of undiagnosed prostate cancers, and this may have attenuated our risk estimates.

In our study, we lack information about the aggressiveness of the disease or information about screening. Aggressive prostate cancer corresponds to a high-risk cancer, meaning it is likely to spread quickly outside the prostate. Although prostate cancer is quite common, almost 15% of all cancers at the time of diagnosis are aggressive.^49^ Given that a percentage of prostate cancer cases are latent, the positive associations observed in our study may reflect increased detection rates due to a higher prevalence of prostate cancer screening that occurs in some subpopulations. Several variables potentially correlated with screening (age, level of education, neighborhood SES index and ethnicity) were included in the models as adjustment variables, mitigating this potential bias to some extent. In our model, adjusting on the neighborhood socioeconomic status index (a composite variable including other variables such as household income, education, employment, living in rental housing or having moved in the last 5 years), overadjustment remains a possibility. We nevertheless considered it in the adjustments since its link with prostate cancer may exist through other known or unknown unmeasured factors. The latency period was chosen in accordance with the natural history of prostate cancer, but this choice may not be ideal. Different approaches were used to estimate air pollutants concentrations and they have some limitations, including the use of six-character postal codes to geolocate the residential addresses. These postal codes are accurate in urban areas but less so in rural areas as they can cover much larger areas (the sensitivity analysis characterized by the exclusion of subjects from rural areas effectively showed higher ORs); with the satellite method, it is assumed that the spatial patterns remained the same from 1975 to 2005 for NO_2_ and from 1975 to 2001 for PM_2.5_ and the resolution was limited to 10 km^2^; by integrating historical data, the calibration of these satellite data has partially addressed this concern; furthermore, concerning scaled PM_2.5_, the poor spatial and temporal coverage of monitoring prior to 1984 led to the use of historical data based on TSP monitoring data and Census Metropolitan Area (CMA) indicator variables. These limitations potentially led to nondifferential misclassification in that the assessments were made independently of the case-control status of the subjects. Finally, although we were able to estimate exposure from residential histories, no information was available for other important microenvironments such as workplaces or transportation.

The study benefits from several strengths. First, we examined a large sample of prostate cancer cases (1,420 cases and 1,424 controls). In addition, the study was based on the population-based enrollment of incident cases and the use of standardized questionnaires to collect information for a wide array of risk factors. We also had detailed information on residential history, which allowed us to construct detailed exposure metrics for an etiologically relevant time period and to assign NDVI levels, which have been inversely associated with ambient PM_2.5_ and also to prostate cancer.^50,51^ We were also able to evaluate associations for two pollutants using exposure derived from both land-use regression and satellite imaging. The former provides a more spatially resolved measure of air pollution,^52^ but findings based on the different approaches allow the impacts of temporality and spatial resolution to be evaluated. For example, a comparison of findings across the three metrics of NO_2_ suggests that, for risk characterization, it is important to capture intra-urban variations in concentrations. Several sensitivity analyses confirmed the robustness of our main findings.

## Conclusions

We found a positive association between air pollution and prostate cancer risk, providing further evidence in support of IARC’s classification of outdoor air pollution as a human carcinogen.^10^ We recognize that few studies to date evaluated the role of air pollution in the development of prostate cancer, and that more work is needed in this area. In light of the study limitations, and of the significant clinical and economic burden of prostate cancer on the population, future research should account for the role of screening in the early detection of prostate cancer, and by extension, characterize variations in risk by prostate cancer aggressiveness.

## Acknowledgments

Leslie Michele-Ange Kouam Youogo gratefully acknowledges Carleton University for the opportunity and the financial support. She also thanks to the entire pedagogical team of the “Institut de santé publique, d’épidémiologie et de développement” (ISPED) of Bordeaux for their teaching and support throughout the realization of this project.

## Conflicts of interest statement

The authors declare that they have no conflicts of interest with regard to the content of this report.

Data analyzed in this article are not publicly available due to the personal identifying information collected in this study.

## Supplementary Material


