# Supplementary material for: Ambient air pollution and prostate cancer risk in a population-based Canadian case-control study
Source: Environ Epidemiol. 2022 Jul 19;6(4):e219. doi: 10.1097/EE9.0000000000000219 (PMC9374191; doi:10.1097/EE9.0000000000000219)
Supplement: Supplementary file 1 [file ee9-6-e219-s001.pdf]

Supplementary information

Appendix

Ambient air pollution and prostate cancer in a population-based Canadian case-control study

Leslie Michele-Ange Kouam [a, b], Marie-Elise Parent [c, d, e], Perry Hystad [f], Paul J Villeneuve [a]

[a] Department of Neuroscience, Carleton University, Ottawa, ON, Canada K1S 5B6
[b] University of Bordeaux, ISPED, Bordeaux, France
[c] Epidemiology and Biostatistics Unit, Centre Armand-Frappier Santé Biotechnologie, Institut national de la recherche scientifique, Université du Québec, Laval, Quebec, Canada
[d] Department of Social and Preventive Medicine, School of Public Health, Université de Montréal, Montreal, Quebec, Canada
[e] Centre de recherche du CHUM, Montreal, Quebec, Canada
[f] College of Public Health and Human Sciences, Oregon State University, Corvallis, OR, USA

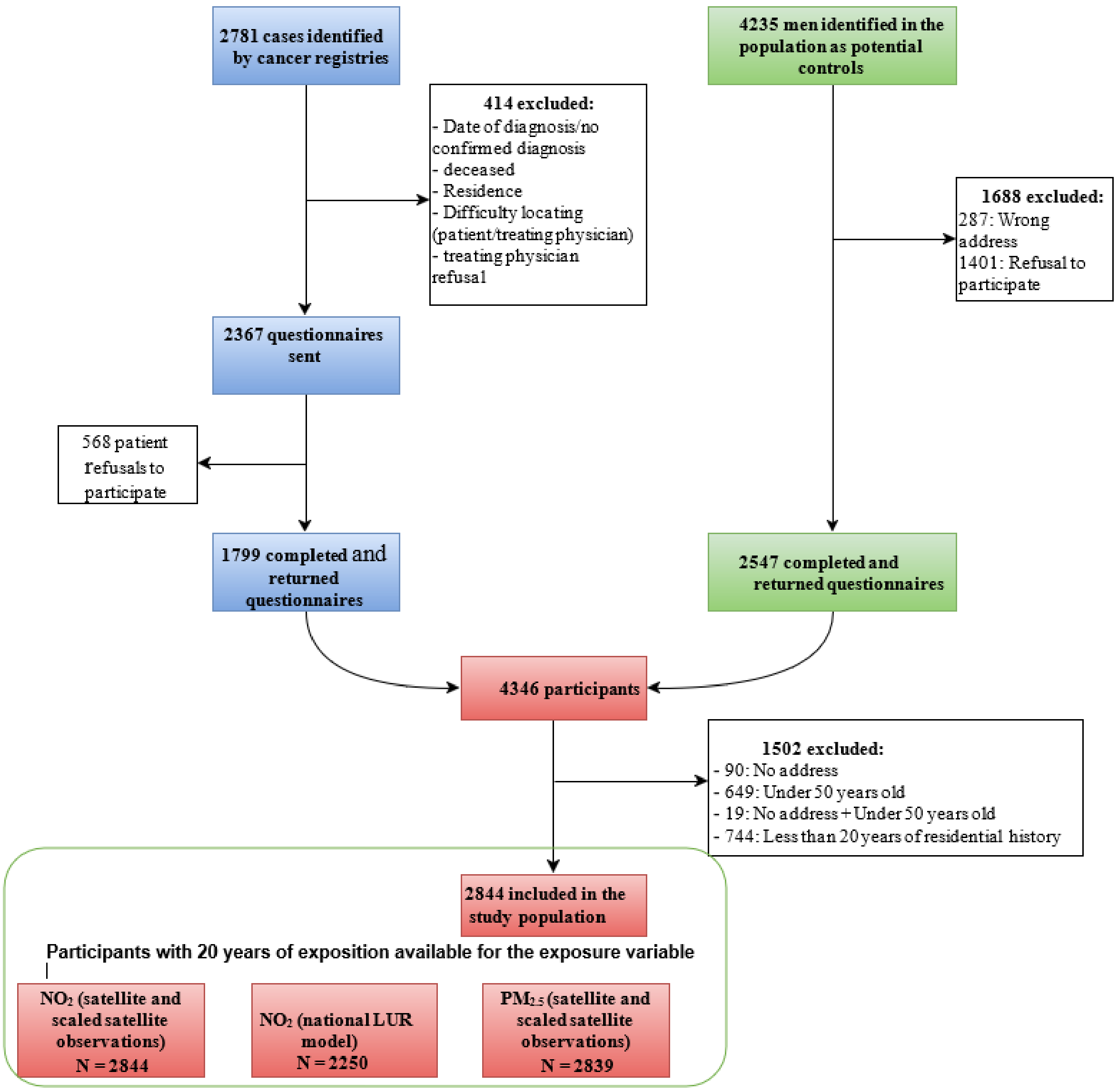

Figure S1. Flow-chart of study population

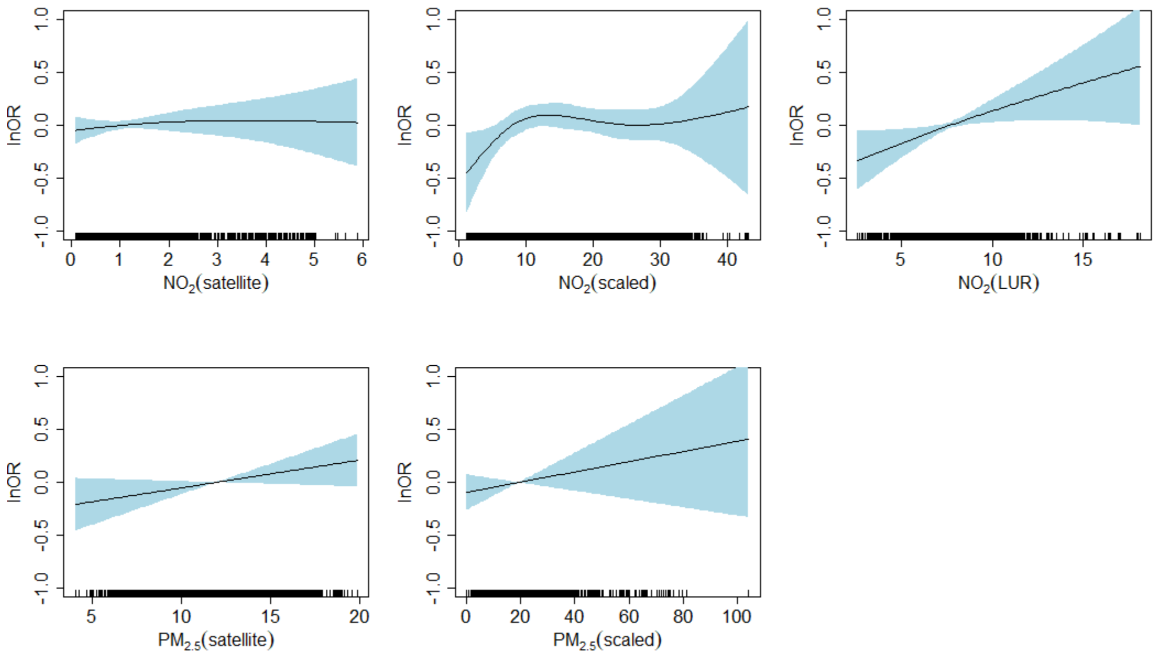

Figure S2a. Exposure-response curves for the relationship between air pollutant concentrations (solid line) and 95% CI (blue shade) over the period from 1975 to 1994 and prostate cancer generated obtained using cubic smoothing splines at 4 df (model 1).

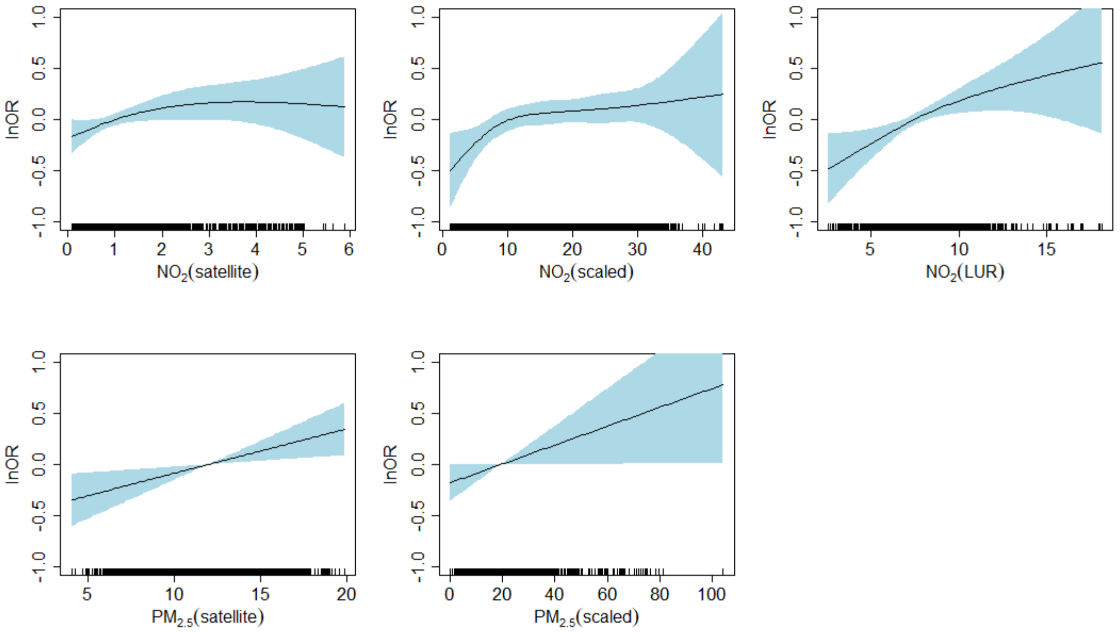

Figure S2b. Exposure-response curves for the relationship between air pollutant concentrations (solid line) and 95% CI (blue shade) over the period from 1975 to 1994 and prostate cancer generated obtained using cubic smoothing splines at 4 df (model 2).

Table S1a. Relationship between prostate cancer risk and having lived near a highway or/and a major road. Unconditional logistic regression on imputed data. Case-control study, NECSS, Canada 1994-1997.

Variable	N	Model 1 * RC (IC 95%)	Model 2 ** RC (IC 95%)	Model 3 *** RC (IC 95%)
**Have lived near a highway and/or major road**	2 844			
– Never		Ref.	Ref.	Ref.
– Others		1.07 (0.91 -1.25)	1.11 (0.94 -1.30)	1.15 (0.97 -1.38)

*Model 1 adjusted for age and province.

**Model 2 adjusted for: age, province, ethnicity, smoking status, alcohol, BMI, years of education, moderate physical activity, strenuous physical activity, total caloric intake, exposure to pesticides and exposure to cadmium

***Model 3 adjusted for: model 2 variables, neighborhood SSE index and residential surrounding greenness.

Table S1b. Relationship between prostate cancer risk and having lived near a highway or/and a major road. Unconditional logistic regression on complete data. Case-control study, NECSS, Canada 1994-1997.

Variable	N	Model 1 * RC (IC 95%)	N	Model 2 ** RC (IC 95%)	N	Model 3 *** RC (IC 95%)
**Have lived near a highway and/or major road**	2 838		2 735		2 031	
– Never		Ref.		Ref.		Ref.
– Others		1.07 (0.91 -1.26)		1.12 (0.95-1.32)		1.09 (0.89-1.32)

*Model 1 adjusted for age and province.

**Model 2 adjusted for: age, province, ethnicity, pack-years, alcohol, BMI, years of education, moderate physical activity, strenuous physical activity, total caloric intake, exposure to pesticides and exposure to cadmium

***Model 3 adjusted for: model 2 variables, neighborhood SSE index and residential surrounding greenness.

Table S2. Association between ambient concentrations of NO_2 and $PM_{2.5}$ around residences and incidence of prostate cancer according to interquartile range increases, 1975-1994 (complete data).

Variables	N	Model 1 * OR (95% CI)	N	Model 2 ** OR (95% CI)	N	Model 3 *** OR (95% CI)
**NO_2 exposure estimates (ppb). for an increase equal to the IQR[a]**						
NO_2 (Satellite)	2,838	1.03 (0.92-1.15)	2,735	1.09 (0.97-1.23)	2,031	1.07 (0.93-1.22)
NO_2 (Scaled)	2,838	1.07 (0.92-1.25)	2,735	1.19 (1.01-1.40)	2,031	1.18 (0.97-1.43)
NO_2 (National LUR)	2,247	1.08 (0.95-1.23)	2,165	1.15 (1.00-1.32)	2,031	1.19 (1.02-1.38)
**$PM_{2.5}$ exposure estimates (µg/m3). for an increase equal to the IQR[b]**						
$PM_{2.5}$ (Satellite)	2,833	1.23 (1.04-1.45)	2,730	1.31 (1.10-1.55)	2,031	1.27 (1.03-1.57)
$PM_{2.5}$ (Scaled)	2,833	1.12 (0.98-1.29)	2,730	1.23 (1.07-1.42)	2,031	1.21 (1.01-1.45)

* Model 1 adjusted for age and province.

** Model 2 adjusted for: age, province, ethnicity, pack-years, alcohol, BMI, years of education, moderate physical activity, strenuous physical activity, total caloric intake, exposure to pesticides and exposure to cadmium

*** Model 3 adjusted for: model 2 variables, neighborhood SSE index and residential surrounding greenness.

IQR[a] =interquartile range: 1.45 ppb for satellite NO_2, 15.18 for scaled NO_2 and 15.39 for national LUR NO_2.

IQR[b] =interquartile range: 3.56 ppb for satellite $PM_{2.5}$ and 4.48 for scaled $PM_{2.5}$.

Table S3. Association between ambient concentrations of NO_2 and $PM_{2.5}$ around residences and incidence of prostate cancer according to interquartile range increases, restricted analysis on those lived in urban areas, 1975-1994 (imputed data).

Variables	N	Model 1 * OR (CI 95%)	Model 2 ** OR (CI 95%)	Model 3 ** OR (CI 95%)
**NO_2 exposure estimates (ppb). for an increase equal to the IQR[a]**				
NO_2 (Satellite)	2 159	1.03 (0.91-1.17)	1.10 (0.97-1.25)	1.10 (0.96-1.26)
NO_2 (Scaled)	2 159	1.11 (0.93-1.32)	1.26 (1.05-1.50)	1.27 (1.05-1.54)
NO_2 (national LUR)	1 897	1.13 (0.98-1.31)	1.21 (1.04-1.40)	1.24 (1.07-1.45)
**$PM_{2.5}$ exposure estimates (µg/m3). for an increase equal to the IQR[b]**				
$PM_{2.5}$ (Satellite)	2 154	1.25 (1.03-1.52)	1.31 (1.08-1.60)	1.31 (1.07-1.60)
$PM_{2.5}$ (Scaled)	2 154	1.12 (0.95-1.31)	1.21 (1.03-1.43)	1.21 (1.02-1.44)

* Model 1 adjusted for age and province.

** Model 2 adjusted for: age, province, ethnicity, pack years, alcohol, BMI, years of education, moderate physical activity, strenuous physical activity, total caloric intake, exposure to pesticides and exposure to cadmium

*** Model 3 adjusted for: model 2 variables, neighborhood SSE index and residential surrounding greenness.

IQR[a] =interquartile range: 1.61 ppb for satellite NO_2, 16.04 for scaled NO_2 and 15.02 for national LUR NO_2.

IQR[b] =interquartile range: 3.59 ppb for satellite $PM_{2.5}$ and 4.28 for scaled $PM_{2.5}$.
